# Lest We Forget

**Published:** 1911-07

**Authors:** 


### "Lest We Forget"

**University of Buffalo, Twenty-fifth Alumni Banquet held April 27, 1900**

(From the *Buffalo Express*, April 28, 1900)

IT was exactly 1.35 o'clock, April 28, when Hon. W. I. Buchanan, Director General of the Pan-American Exposition, got on his feet in the big dining room at the Iroquois and said:

"I propose never again to allow a medical man to say anything to me about the evils of late dining."

The occasion was the annual banquet of the Alumni Association of the Medical Department of the University of Buffalo, and Mr. Buchanan's remarks were addressed to an audience composed of some 150 physicians, who appreciated them thoroughly.

It was about as late a banquet as one would care to attend, but the excellence of the after-dinner utterances amply repaid one for lost sleep. There were seven speakers on the toast list and it is an extremely rare occasion when seven as good after-dinner speeches are made in one evening.

Dr. William Warren Potter, president of the association, and who delivered the Alumni address at the inaugural meeting of the association held February 28, 1875, twenty-five years before, acted as toastmaster, and at 12.18 o'clock he rapped for order and the physicians lighted their cigars and listened.

He said:

"The executive committee has asked me simply to preside but I find myself loaded with the responsibility of conducting the ceremonies."

Taking for his text the figures on the menu, 1875-1900, he continued:

TWENTY-FIVE YEARS!

What memories such a period of time enkindles! What trials it suggests! What triumphs it records! What disaster it engulphs! What achievements it makes possible! !

And yet 'tis but a breath. With a touch of the Infinite it vanishes into eternity, fading "like a wreath of mist at eve" as millions of similar periods have done, "to darkle in the trackless void" made and ever making by remorseless time.

Twenty-five years! !

Within that period a child is born, passes through infancy, childhood and youth, goes through college, takes his place in community, and begins his life work. This, in brief, is the history of young individual life. In national life it is much the same. Territories are peopled, states are formed and before we realise it, senators and representatives in congress are debating over systems of education, methods of internal improvement, and other great problems of statehood for the new commonwealth.

Twenty-five years ago, when this association was organised, the University, whose 54th annual commencement we have witnessed tonight, was located well up-town at Main and Virginia Streets, almost on the border of a city of 100,000 inhabitants. Now on the summit of yonder hill at High Street, stands a magnificent building, located near the center of a prosperous city, the second in importance within the state, with 400,000 people who are busy with manufactures, arts, commerce, science and all the varied and various interests that concern this progressive period; and, are, withal at this very moment preparing to entertain within the next year all the nations of the western hemisphere, who will assemble here at a great exposition.

A quarter of a century ago there were seven professorial chairs embracing the principal departments of medicine, with, perhaps, a few clinical assistants; now, behold a great college, with its 70 professors, teachers and instructors. Then there were no laboratories of biology, bacteriology, pathology, physiology or chemistry, or perhaps only the faintest semblance of the two latter. Now all these are in full swing under the best of teachers.

In those days clinical teaching had not reached its present state of perfection; indeed, the didactic method was paramount, and the dictum of the professor was the pupil's authority. Now, bedside instruction constitutes the larger and better portion of the curriculum. The course then was of two years' duration, with no uniform requirements of preliminaries, and no license by the state was demanded. The course now is of four years and graded, the preliminaries must equal the acquirements of a high school graduate, and there are separate state examinations for license to practise.

Finally, in those days there were no women pupils and no alumnæ, while now there are more than 100 of the latter, who are well represented here this evening, every one a credit to Alma Mater.

It must not be inferred from what I have said that Buffalo University Medical College was not in those days a strong, well equipped school, for, in reality, it was one of the best and fully abreast of the period in its methods of instruction. It was founded by some of the ablest men in the country, and the founders have been succeeded from time to time as they disappeared from the scene, by men of great learning, skill and profundity. If they did not ask as much of candidates for medical degrees as your present teachers, it was because nowhere was as much required as in this busy bustling age of iconoclasm—this iron age of positivism.

For obvious reasons, I may not speak of the individual members of the present teaching body. Many of them are grouped around these tables and within the sound of my voice. They are well known to you all; they have your confidence and esteem, as they have that of the citizens of this community. They are distinguished among the profession of this country as teachers and practisers of the various sciences and arts pertaining to medicine and surgery; indeed, their fame is not limited by the borders of American shores, but has gone abroad wherever legitimate medicine is recognised throughout the world.

The class of 1900 is fortunate, most fortunate, in the choice of its environment for entrance into the most humane and ennobling of the learned professions.

If, then, I may not speak individually of your faculty, I trust I may be pardoned for making personal reference to one of the number, one who is loved and respected by you all, as he is also by this entire community, one who has been greviously afflicted during the past few weeks with direful sickness, but who, happily, is now to all appearances on the high road to speedy recovery. I ask you, one and all, to rise in your places and drink his health, sending him words of greeting and best wishes for his early recovery, and for a long and prosperous life. Ladies and gentlemen, I pledge you the health of Professor Roswell Park.

The response was prompt, the audience rose en masse and amidst rousing cheers, flying napkins, and rattling glasses, the health of Dr. Park was eagerly drunk.

The toastmaster, paying little heed to the printed rotation, then announced the sentiments and called upon the speakers in the following order of sequence:

Alma Mater . . . . . . . . . . . . . . . . . . . . . . . Dr. Charles G. Stockton

"He thought as a sage, though he felt as a man."

The Class of '00 . . . . . . . . . . . . . . . . . . . Dr. William Fred. Powers

"Think naught a trifle, though it small appear."

The Physician Idealised . . . . . . . . . . . . . . Dr. Cora Billings Lattin

"It were a journey like the path of heaven,
To help you find them."

Preaching and Practising . . . . . . . . . . . . Rev. George B. Richards

The Pan-American, Hon. William I. Buchanan, Director-General

"Lest men suspect your tale untrue,
Keep probability in view."

The Ohio Idea . . . . . . . . . Dr. Charles A. L. Reed, Cincinnati, Ohio

"There is occasions and causes why and wherefore
in all things."

Dr. Potter, who made an excellent toastmaster, then introduced Professor Charles G. Stockton, who, in response to the sentiment "Alma Mater" pointed out that while the average resources of the medical schools of the country are not great, the University of Buffalo is exceptionally well equipped in many respects. In spite of that fact, however, the University needs help, and he appealed to the new graduates to help it all they could.

Dr. William Fred Powers, responding to the sentiment "The Class of '00" delivered a neat appreciation of the efforts of the faculty which, he said, had had the result that the members of the class just graduated knew more about the practice of medicine than anyone else in the world.

Extremely bright and clever was the response of Dr. Cora Billings Lattin to the toast "The Physician Idealised." She appealed for wider recognition, by the male members of the profession, of the value and need of the woman physician and pointed out that, besides filling a real need in the life of suffering womankind, the woman doctor could do much towards purging medical ethics of many characteristics which are undesirable.

Rev. George B. Richards, responding to the toast "Preaching and Practising," made an appeal for the admission of the clergy into sick rooms. "If the preacher is a fool," he said, "keep him out, just as you would a fool doctor, but recognise the fact that a wise clergyman can often be of real benefit to your patient."

Director General Buchanan spoke on "Reciprocity in Medicine." He declared that the South American republics today offer the best field in the world for the American physician who can talk Spanish, and he told of efforts that are being made to effect an interchange of courtesies that will secure the recognition of American diplomas in those countries.

Dr. Charles A. L. Reed, of Cincinnati, chairman of the executive committee of the Pan-American Medical Congress, who followed Mr. Buchanan, confirmed his statement regarding the value of South America as a field for practice, and offered to give all those present such credentials as would insure them a foothold in any of the Latin-American republics. Then, in a witty reply to Mr. Richards, he pointed out that the danger of allowing ministers in the sick room was that they might induce the patient to try some of the patent medicines which the clergy are so fond of indorsing for advertising purposes.

At 2.10 A. M., the toastmaster, Dr. William Warren Potter, thanked the speakers and the audience who had so ably responded to the sentiments of the evening and so patiently listened thereto, thus materially aiding the executive committee in its efforts to make the occasion interesting. He then declared the meeting adjourned.

At the commencement exercises of the university, which were held in Central Presbyterian Church before the banquet, Dr. Reed made the principal address, dealing with the history of "Folk Medicine" and the development of modern medical science.

(Note.—In the light of recent events, the preceding is republished trusting it may prove of interest to some if not to all of the members of the Alumni Association of the University of Buffalo.—The Editor.)

